# Insufficient free-time physical activity and occupational factors in Brazilian public school teachers

**DOI:** 10.1590/S1518-8787.2017051006217

**Published:** 2017-07-07

**Authors:** Douglas Fernando Dias, Mathias Roberto Loch, Alberto Durán González, Selma Maffei de Andrade, Arthur Eumann Mesas

**Affiliations:** I Programa de Pós-Graduação em Saúde Coletiva. Universidade Estadual de Londrina. Londrina, PR, Brasil; II Departamento de Educação Física. Universidade Estadual de Londrina. Londrina, PR, Brasil; III Departamento de Saúde Coletiva. Universidade Estadual de Londrina. Londrina, PR, Brasil

**Keywords:** Faculty, Education, Primary and Secondary, Working Conditions, Sedentary Lifestyle, Motor Activity, Occupational Health

## Abstract

**OBJECTIVE:**

To evaluate if perceived occupational factors are associated with insufficient free-time physical activity in Brazilian public school teachers.

**METHODS:**

The relationship between insufficient physical activity (< 150 minutes/week) and variables related to work was analyzed in 978 elementary and high school teachers calculating the prevalence ratio (PR) and 95% confidence interval (95%CI) in Poisson regression models, adjusted for sociodemographic and health variables.

**RESULTS:**

The prevalence of insufficient physical activity was 71.9%, and this condition was associated independently with the perception of bad or regular balance between personal and professional life (PR = 1.09; 95%CI 1.01–1.18), perception that standing time affects the work (PR = 1.16; 95%CI 1.01–1.34), low or very low perception of current ability for the physical requirements of work (PR = 1.21; 95%CI 1.08–1.35), and temporary employment contract (PR = 1.13; 95%CI 1.03–1.25). The teaching of physical education was associated with lower prevalence of insufficient physical activity (PR = 0.78; 95%CI 0.64–0.95).

**CONCLUSIONS:**

The perception of adverse working conditions is associated with increased prevalence of insufficient physical activity in teachers and should be considered for the promotion of physical activity in this population.

## RESUMO

**OBJETIVO:**

Analisar se fatores ocupacionais percebidos estão associados à atividade física insuficiente no tempo livre em professores de escolas públicas.

**MÉTODOS:**

A relação entre atividade física insuficiente (< 150 minutos/semana) e variáveis relacionadas ao trabalho foi analisada em 978 professores do ensino fundamental e médio mediante o cálculo da razão de prevalência (RP) e intervalo de confiança de 95% (IC95%) em modelos de regressão de Poisson, ajustados por variáveis sociodemográficas e de saúde.

**RESULTADOS:**

A prevalência de atividade física insuficiente foi de 71,9%, e essa condição associou-se de maneira independente com percepção de equilíbrio entre vida pessoal e profissional ruim ou regular (RP = 1,09; IC95% 1,01–1,18), percepção de que o tempo de permanência em pé afeta o trabalho (RP = 1,16; IC95% 1,01–1,34), percepção de capacidade atual para as exigências físicas do trabalho baixa ou muito baixa (RP = 1,21; IC95% 1,08–1,35) e contrato de trabalho temporário (RP = 1,13; IC95% 1,03–1,25). Ministrar disciplina de educação física associou-se com menor prevalência de atividade física insuficiente (RP = 0,78; IC95% 0,64–0,95).

**CONCLUSÕES:**

A percepção de condições de trabalho negativas associa-se à maior prevalência de atividade física insuficiente em professores e devem ser consideradas para a promoção de atividade física nessa população.

## INTRODUCTION

Studies conducted in different regions of the world have pointed out that occupational factors may affect the health status of teachers^1,2,17,21^. In this sense, such characteristics may also negatively affect the practice of physical activity, especially when teachers need to use their free-time to perform work tasks. Among the few studies on this subject, most have investigated teachers of specific subjects such as, for example, physical education^6^.

We have found only one study^5^ that has focused on the relationship between physical activity and occupational factors in teachers of different areas of knowledge. The study has been carried out with teachers from Belgium and free-time physical activity has been associated with better job satisfaction and lower levels of occupational stress and absenteeism^5^. However, the researchers have addressed physical activity as an independent variable and they have not analyzed specific issues of the teaching work that could affect the practice of free-time physical activity, such as total teaching time, type of employment contract, indicators of physical effort at work, among others.

In Brazil, we believe that the effect of the working conditions on the practice of physical activity can be even more pronounced when compared to other countries. A research report recently conducted with teachers from 34 countries has shown that, on average, Brazil presents a greater number of students per class and lower prevalence of teachers with permanent contract; in addition, teachers have increased weekly hours dedicated to teaching^a^. Students can have classes in the morning, afternoon, and night, in this way a certain portion of teachers seeks to supplement their income working on up to three shifts or even in different jobs, thereby reducing their free-time.

The prevalence of insufficient physical activity in Brazilian teachers varies between 34%^15^ and 75%^11^, and some studies^7,19^ have found values close to those observed for the adult Brazilian population, of 48.7%^b^. Although the frequency of such behavior is not especially higher in teachers, the social relevance of teaching activities and the possibility that specific aspects of this work are involved in low adherence to healthy behaviors reinforce the need for the problem to be studied in this professional category. In this sense, some occupational factors are associated with low levels of physical activity in the general population^8,12^, but this approach has not been applied to teachers.

Therefore, the objective of this study has been to evaluate the association of occupational factors and insufficient free-time physical activity in public school teachers. We aim to examine the hypothesis that some occupational factors are associated with difficulties to perform free-time physical activity, which can contribute to both broaden the scientific knowledge on this issue and to subsidize the planning of more effective interventions to fight the low adherence to this behavior among these professionals.

## METHODS

This cross-sectional observational study is part of a research project (PRÓ-MESTRE) developed in the Graduate Program in Public Health at the Universidade Estadual de Londrina, whose overall objective is to assess the health status and lifestyle of public school teachers and relate them with aspects of the work process.

Data collection took place between August 2012 and June 2013. Initially, all 73 state public schools in the city of Londrina, State of Paraná, Brazil, were ordered according to the number of teachers (data provided by the Regional Education Center of Londrina). Then all teachers working in the classroom, in the elementary and high school levels, of the 20 largest schools (which had 70 or more teachers), were invited to participate in the study. The data were obtained from pre-scheduled interviews conducted outside the class period of the teachers by previously trained interviewers. The interviews lasted approximately 40–50 minutes and were followed by the completion of a questionnaire, with approximate duration of 10 to 20 minutes.

Insufficient free-time physical activity, henceforth abbreviated as IPA, was the dependent variable in this study. Although some studies use the name “leisure time” physical activity, we decided to use the expression “ free-time” to designate clearly all the time not occupied with work issues, which may not necessarily be for leisure. To obtain this information, the participants first answered if in a typical week they performed some type of free-time physical activity. If so, they described what were the activities, the weekly frequency, and duration in minutes.

After collecting the data, the physical activities mentioned by the teachers were classified according to intensity in metabolic equivalent of task (MET), into light (< 3 MET), moderate (3 to 6 MET), or vigorous (> 6 MET). The parameter used to establish the MET was based on the Portuguese version of the Compendium of Physical Activities^10^. Then, we calculated the weekly time in minutes spent in physical activities. In this step, light activities were disregarded, and the time spent in vigorous activities was multiplied by two. Finally, after identifying the time in minutes of the weekly physical activity of every teacher, we classified them into three categories: 1) inactive in free-time (0 to 9 minutes per week), 2) insufficiently active in free-time (10 to 149 minutes per week), and 3) active in free-time (150 minutes or more per week). For the crude and adjusted prevalence ratios (PR) between the physical activity level and occupational factors, categories 1 and 2 were grouped operationally in order to form a category with only individuals who did not reach 150 minutes of IPA^14^.

The independent variables and their respective categories were: a) time of profession (0 to 4 years, 5 to 9 years, 10 to 19 years, ≥ 20 years); b) number of shifts they teach (one, two, three); c) type of employment contract (temporary, statutory); d) weekly hours as a teacher (< 21 hours, 21–40 hours, > 40 hours); e) subject they teach (physical education, other); f) perception regarding the balance between personal and professional life (excellent, good, regular, bad); g) perception that standing time affects the work (affects, does not affect); h) perception regarding the current ability to work in relation to physical requirements (very good, good, moderate, low, very low); i) frequency that they consider having enough time to fulfill all the work (often, sometimes, never or almost never, rarely); j) frequency that they feel exhausted at the end of the workday (never, a few times a year, a few times a month, a few times a week, daily); and k) frequency that they feel exhausted when getting up after a night’s sleep to face a workday (never, a few times a year, a few times a month, a few times a week, daily).

We also obtained information about the following variables of adjustment (and their respective categories): a) gender (male, female); b) age group (≤ 29 years, 30 to 39 years, 40 to 49 years, 50 to 59 years, ≥ 60 years); c) monthly family income (R$600.00 to R$3,000.00, R$3,001.00 to R$5,000.00, R$5,001.00 to R$7,000.00, > R$7,000.00); d) perception regarding their health (excellent, very good, good, bad, very bad); e) body mass index (BMI) in kg/m^2^ (< 25, 25 to 29.9, ≥ 30) calculated from self-reported weight and height; and f) presence of chronic pain, i.e., report of painful symptoms for six months or more (yes, no). The definition of the cutoff points for the income ranges was based on expected possible answers, without following any pre-defined criteria.

The forms were double-typed using the program EpiInfo, version 3.5.2 for Windows, and, later, we compared both data files and corrected the errors detected. For the descriptive analysis, we used the absolute and relative frequency distribution, while for the bivariate analysis we used the Chi-square test. The crude and adjusted PR of the IPA according to the types of occupational factors were obtained by Poisson regression with robust variance adjustment. Two models were built for each occupational factor evaluated (example: type of employment contract, number of shifts, workload, etc.), seeking to analyze their relationship with IPA (dependent variable). To evaluate whether such associations were independent of potential confounding factors, the adjustment was carried out in two stages, considering two factors: socio-demographic and health variables. The first model adjusted the association of interest by gender, age, and monthly family income (sociodemographic variables), and the second one included, in addition to the variables of the first model, perceived health, body mass index, and presence of chronic pain (health status variables). This progressive adjustment aimed to allow the observation of the possible confounding effect exclusively caused by the sociodemographic characteristics and, as a result, the additional effect that could be contributed by the health status. For example, if the associations of interest were initially statistically significant and then lost their significance only in the second model, we could identify that the confounding factors were the variables of the health status (and not the sociodemographic ones). The inclusion of these variables in the models followed the theoretical criterion, namely, the existence of evidence in the literature that could confound the associations of interest because they are related to both physical activity and work. Data analyses were performed in the program Stata, version 9 for Windows. Additional analyses were performed by repeating all models with the dependent variable of practice of free-time physical activity (yes or no), as the results could be different when compared with the variable IPA adopted in the main analyses.

This study was approved by the Research Ethics Committee of the Universidade Estadual de Londrina (CAAE: 01817412.9.0000.5231) and all participants signed the informed consent.

## RESULTS

Among the 1,126 teachers considered as eligible for the study, 63 (5.6%) refused to participate, 65 (5.8%) were on medical leave, and 20 (1.8%) were not located after five attempts. Thus, the final sample consisted of 978 teachers (86.9%).

Table 1 describes the socio-demographic and health characteristics evaluated. Among the 978 respondents, most were women (69%), aged up to 49 years (average age: 41.5 years; SD = 10.0 years) (79.2%), had monthly family income of up to R$5,000.00 (59.5%), and had BMI ≥ 25 kg/m^2^ (52.0%). Additionally, 42% reported feeling chronic pain. Regarding work characteristics, 31% of the teachers had temporary employment, 16% worked in three shifts (morning, afternoon, and evening), and 21% worked as a teacher for more than 40 hours per week (Table 2).

**Table 1:** free-time physical activity according to socio-demographic and health variables in teachers. Londrina, State of Paraná, Brazil, 2012–2013.

Variable	n	free-time physical activity				

		%	Inactive^a^ (%)	Insufficient^b^ (%)	Active^c^ (%)	p^d^
Total	978	100	55.7	16.2	28.1	
Gender						0.002
Female	670	68.5	59.1	17.0	23.9	
Male	308	31.5	48.4	14.3	37.3	
Age group (years)						0.665
< 30	130	13.3	57.7	12.3	30.0	
30–39	295	30.2	59.0	12.9	28.1	
40–49	330	33.7	53.3	17.9	28.8	
50–59	183	18.7	55.2	19.1	25.7	
> 59	40	4.1	47.5	25.0	27.5	
Monthly family income (R$)^e,f^						0.071
< 3,001.00	241	24.9	59.8	13.3	27.0	
3,001.00 to 5,000.00	335	34.6	56.4	16.4	27.2	
5,001.00 to 7,000.00	239	24.7	54.8	17.6	27.6	
> 7,000.00	154	15.9	49.4	16.9	33.8	
Perceived health^f^						
Very good/Excellent	346	36.4	47.4	16.5	36.1	<0.001
Good	557	58.6	60.1	16.2	23.7	
Bad/Very bad	47	4.9	63.8	14.9	21.3	
Body mass index (kg/m^2^)^f^						
> 30.0	163	16.8	65.0	18.4	16.6	0.003
25.0–29.9	342	35.2	54.1	16.4	29.5	
< 25.0	466	48.0	53.2	15.5	31.3	
Chronic pain^f^						
Yes	411	42.2	60.3	16.3	23.4	0.014
No	562	57.8	52.3	16.0	31.7	

^a^ Inactive in free-time = zero to 10 minutes per week.

^a^ Insufficiently active in free-time = 11 to 149 minutes per week.

^a^ Sufficiently active in free-time = 150 minutes or more of physical activity per week.

^d^ Chi-square test.

^3^ Average value of Real in the period of data collection: US$1.00 = R$2.00.

^f^ Total number of participants was lower than 978 because of missing information for the respective variable.

**Table 2:** Free-time physical activity according to occupational variables in teachers. Londrina, State of Paraná, Brazil, 2012–2013.

Variable	N	Free-time physical activity				

		%	Inactive^a^ (%)	Insufficient^b^ (%)	Active^c^ (%)	p^d^
Time working in the profession (years)^e^						0.513
< 5	174	17.8	56.9	14.4	28.7	
5–9	218	22.3	56.9	15.6	27.5	
10–19	287	29.3	59.2	12.2	28.6	
≥ 20	299	30.6	50.8	21.4	27.8	
Number of shifts						0.676
1	188	19.2	56.9	17.6	25.5	
2	628	64.2	55.3	15.9	28.8	
3	162	16.6	56.2	15.4	28.4	
Type of employment						0.024
Statutory	306	31.3	52.8	17.0	30.2	
Temporary	672	68.7	62.1	14.4	23.5	
Working hours as a teacher (hours/week)						0.359
< 21	163	16.7	58.3	12.3	29.4	
21–40	611	62.5	55.8	17.8	26.4	
> 40	204	20.9	53.4	14.2	32.4	
Subject taught						0.001
Other	893	91.3	57.2	16.2	26.5	
Physical Education	85	8.7	40.0	15.3	44.7	
Perception of balance between personal and professional life						0.013
Good/Excellent	704	72.0	53.3	16.1	30.7	
Bad/Regular	274	28.0	62.0	16.4	21.5	
Perception of how long standing affects the work						0.007
Does not affect	148	15.1	44.6	17.6	37.8	
Affects	830	84.9	57.7	15.9	26.4	
Perception of the current ability to work in relation to physical requirements^e^						0.001
Good/Very good	588	60.2	53.2	13.8	33.0	
Moderate	324	33.2	58.0	20.1	21.9	
Low/Very low	65	6.7	66.2	18.5	15.4	
Frequency that they consider having enough time to fulfill all the work^e^						0.255
Frequently	166	17.5	50.6	19.3	30.1	
Sometimes	442	46.5	55.9	16.1	28.1	
Never or almost never/Rarely	342	36	57.6	15.2	27.2	
Frequency that they feel exhausted at the end of the workday^e^						0.037
Never/A few times a year	134	14.1	50.7	17.2	32.1	
A few times a month	200	21.1	49.5	19.5	31.0	
A few times a week/Daily	613	64.7	58.6	15.0	26.4	
Frequency that they feel exhausted when getting up after a night’s sleep to face a workday^e^						0.071
Never/A few times a year	386	40.7	52.1	17.1	30.8	
A few times a month	254	26.8	57.9	14.2	28.0	
A few times a week/Daily	308	32.5	58.1	16.9	25.0	

^a^ Inactive in free-time = zero to 10 minutes per week.

^a^ Insufficiently active in free-time = 11 to 149 minutes per week.

^a^ Sufficiently active in free-time = 150 minutes or more of physical activity per week.

^d^ Chi-square test.

^e^ Total number of participants was lower than 978 because of missing information for the respective variable.

The prevalence of IPA was 71.9%, with variations according to socio-demographic and health aspects (Table 1) and occupational aspects (Table 2). We observed a higher proportion of IPA among women, those who reported chronic pain, with higher levels of BMI, and those with worse health status. The proportion of IPA was similar in all age and income groups.

Table 3 shows the crude and adjusted PR of the IPA for the categories of occupational exposures. After adjustment for the socio-demographic and health variables, IPA was associated with perception of regular or bad balance between personal and professional life, perception that standing time affects work, low or very low perception of current ability for the physical requirements of work, and temporary employment contract. The teaching of physical education was associated with lower prevalence of IPA.

**Table 3:** Prevalence and crude and adjusted prevalence ratios for insufficient free-time physical activity according to occupational factors in teachers. Londrina, State of Paraná, Brazil, 2012–2013.

Variable	n	IPA (%)	Crude analysis		Model 1^a^		Model 2^b^	
		
			PR	95%CI	PR	95%CI	PR	95%CI
Time working in the profession (years)^c^								
< 5	170	71.8	1		1		1	
5–9	221	71.9	1.00	0.88–1.14	1.00	0.88–1.13	0.99	0.87–1.13
10–19	285	71.2	0.99	0.88–1.12	0.95	0.84–1.08	0.93	0.81–1.06
≥ 20	301	72.4	1.01	0.90–1.13	0.93	0.80–1.08	0.91	0.78–1.07
Number of shifts								
1	188	74.5	1		1			1
2	628	71.2	0.96	0.87–1.05	0.96	0.87–1.06	0.96	0.87–1.06
3	162	71.6	0.96	0.85–1.09	0.98	0.86–1.12	0.98	0.86–1.12
Type of employment								
Statutory	306	69.8	1		1		1	
Temporary	672	76.5	1.10	1.01–1.19^d^	1.12	1.02–1.22^d^	1.13	1.03–1.25^e^
Working hours as a teacher (hours/week)								
< 21	163	70.6	1		1		1	
21–40	611	73.7	1.04	0.97–1.27	1.06	0.95–1.19	1.10	0.98–1.23
> 40	204	67.7	0.96	0.84–1.10	1.01	0.88–1.15	1.02	0.89–1.18
Subject taught								
Other	894	73.4	1		1		1	
Physical Education	84	56.0	0.76	0.63–0.93^e^	0.77	0.63–0.93^e^	0.78	0.64–0.95^d^
Perception of balance between personal and professional life								
Good/Excellent	704	69.3	1		1		1	
Bad/Regular	274	78.5	1.13	1.05–1.23^e^	1.12	1.04–1.21^e^	1.09	1.01–1.18^d^
Perception of how long standing affects the work								
Does not affect	148	62.2	1		1		1	
Affects	830	73.6	1.18	1.04–1.35^d^	1.17	1.03–1.34^d^	1.16	1.01–1.34^d^
Perception of the current ability to work in relation to physical requirements^c^								
Good/Very good	588	67.0	1			1	1	
Moderate	324	78.1	1.17	1.07–1.26^f^	1.14	1.05–1.24^e^	1.09	1.00–1.19^d^
Low/Very low	65	84.6	1.26	1.12–1.42^f^	1.22	1.08–1.38^e^	1.21	1.08–1.35^e^
Frequency that they consider having enough time to fulfill all the work^c^								
Frequently	166	69.9	1		1		1	
Sometimes	442	71.9	1.03	0.92–1.16	1.03	0.92–1.16	1.01	0.90–1.13
Never or almost never/Rarely	342	72.8	1.04	0.92–1.17	1.05	0.93–1.18	1.00	0.89–1.13
Frequency that they feel exhausted at the end of the workday^c^								
Never/A few times a year	134	67.9	1		1		1	
A few times a month	200	69.0	1.02	0.88–1.18	1.02	0.88–1.18	1.01	0.87–1.17
A few times a week/Daily	613	73.6	1.08	0.96–1.23	1.07	0.94–1.21	1.03	0.90–1.17
Frequency that they feel exhausted when getting up after a night’s sleep to face a workday^c^								
Never/A few times a year	386	69.2	1		1		1	
A few times a month	254	72.1	1.04	0.94–1.15	1.04	0.94–1.15	1.01	0.92–1.12
A few times a week/Daily	308	75.0	1.08	0.99–1.19	1.06	0.97–1.17	1.02	0.93–1.12

IPA: insufficient free-time physical activity

^a^ Adjusted by age, gender, and monthly family income.

^b^ Adjusted by age, gender, monthly family income, body mass index, perceived health, and chronic pain.

^c^ Total participants was lower than 978 because of missing information in the database.

^d^ p < 0.05

^e^ p < 0.01

^f^ p < 0.001

The additional analyses, which considered the practice of free-time physical activity as the dependent variable (yes or no), showed results similar to those observed in the main analyses.

## DISCUSSION

This cross-sectional epidemiological study identified a high prevalence of IPA in public school teachers of a Brazilian municipality. Such outcome, even after adjusting for the demographic, economic, and health variables, remained associated with occupational factors, indicating that the prevalence of IPA is higher among teachers who had a worse evaluation or perception of their work conditions and hours.

The prevalence of teachers who did not reach 150 minutes per week of free-time physical activity in this study (71.9%) is above that found by VIGITEL (2014) (65%) in the general population^c^. Although the cutoff point is the same, some specific characteristics of the population of teachers can explain this difference. For example, among the teachers analyzed, we found seven women for every three men, a proportion considerably higher than that observed in the general population^d^. Considering that the level of free-time physical activity is higher among men^c^, it is possible that the composition of the sample according to gender can partially explain the higher prevalence of IPA.

The prevalence of IPA was greater among those who had a worse evaluation of the balance between personal and professional life. Considering that excess of work is one of the main barriers to the practice of free-time physical activity among workers^20^, an explanation for this result is that teachers who assessed negatively the balance between personal and professional lives have less time to practice physical activity, because they use part of the already reduced free-time to perform professional activities, such as preparing lessons and grading papers. This hypothesis is reinforced when we consider that, in the same database, the proportion of teachers who reported having enough time to take care of their health was significantly higher among those who assessed positively (17.1%) the balance between personal and professional life when compared to those who evaluated negatively (5.9%). The balance between personal and professional life is important not only for facilitating health care, but also because of its relationship with quality of life^13^.

The type of employment contract was associated with IPA, so that the prevalence was higher among temporary when compared to statutory teachers. Although we have found no study that compared physical activity among teachers with different types of employment contract, Milani and Fiod^16^ have pointed out that the fewer guarantees of labor rights for temporary workers highlights the precarious work process among teachers. In this sense, we believed that this precariousness could reflect negatively on health indicators, such as the practice of physical activity. Another factor that may explain the higher prevalence of IPA among temporary teachers is their work in places often rejected by teachers with statutory employment. For example, at the beginning of the school term, statutory teachers can choose the schools and classes in which they want to work and, therefore, temporary teachers end up having to take the remaining schools and classes which usually are the most geographically distant or with worse working conditions. However, despite the opportunity to choose schools and classes, the stability, and guarantee of labor rights, statutory teachers are also exposed to the process of precariousness of the work^16^ and, according to the results of this research, they also show high prevalence of IPA.

The IPA was associated with the perception that the time which they remain standing affects their performance at work. Considering that standing for too long is connected with venopathies of lower limbs^4^, the first hypothesis to explain such an association is that teachers who spend more time on foot feel more pain and, as a result, are less physically active during their free-time. However, when such an association was adjusted by the health variables, there was no significant modification in the value of PR, suggesting that the presence of chronic pain does not sufficiently explain, in this sample, such an association. Another hypothesis for this finding is that teachers feel more tired when standing longer to practice free-time physical activity. In addition, they can consider that they had already performed enough physical activity at work and, therefore, it would be unnecessary to practice more. Finally, it is possible that the association with IPA may have occurred in the opposite direction. For example, because they practice physical activity, some teachers may have greater physical fitness to support physiological workloads and, therefore, feel the effect of standing time with less intensity on the performance of teaching activities.

Although the proportion of IPA was lower among physical education teachers when compared to those of other subjects (56% versus 73.4%), it is worth remembering that more than half of that group has not reached the cutoff point of 150 minutes/week. Physical education teachers, probably, make up the professional category which has the greater knowledge about the benefits of this behavior. From this point of view, this result confirms the literature that indicates that knowledge, when isolated from social, environmental, political, economic, and cultural actions, among others, is insufficient for the promotion of physical activity in society^18^. The explanation for the lower prevalence of IPA among physical education teachers can involve both personal aspects, such as motivation or affinity for this practice, and aspects related to the profession, such as feeling the need to be a model for the students and concern with maintaining adequate levels of physical fitness for practices in class^9^.

An important point to be highlighted is that the way in which the characteristics of the teaching work hinder the practice of free-time physical activity can be different according to the variable investigated. For example, factors such as work during off-hours (negative evaluation of the balance between personal and professional lives) hinder the practice of free-time physical activity as the teacher becomes too busy to practice physical activity. In addition, factors that signal excess of work (perception that standing time affects the work) hinder the practice of physical activity, especially because it leaves the teacher physically tired. In this perspective, the strategy to be adopted to increase the level of physical activity of teachers can be more effective when considering the specificities of each factor. However, we must consider that physical activity is a complex behavior and its regular practice is determined by multiple factors (intrapersonal, interpersonal, physical environment, etc.)^3^, so that interventions based on ecological models that consider the characteristics of work together with other factors will, possibly, be more effective than those based only on occupational variables^18^.

Some methodological considerations must be highlighted. Although the associations found may be a result of reverse causation, cross-sectional studies are especially indicated when the objective is to investigate associations between various factors and an outcome, as in this case. Regarding the population studied, the selection of the biggest schools followed a criterion of convenience, to facilitate data collection, and contemplated the totality (ensuring representativeness) only of active teachers in the largest state schools of the city, whose work characteristics might differ from smaller schools. Information on physical activity was not obtained with a validated questionnaire, but it was based on questions that allowed us to characterize the report of the interviewee in relation to the type, frequency, and duration of the free-time physical activities of a usual week. We should also consider that the prevalence of IPA can be higher than what we have found, as workers with worse health status or who perceive more negatively their working conditions tend to have greater amount of sick leaves or even early career abandonment (healthy worker bias). Given the relevance of the subject and the reduced number of works on the habits and behaviors related to health in this population, the strength of this study was the evaluation of physical activity and several occupational factors in a large number of public school teachers. In addition, we positively highlight the low rate of losses and refusals.

We conclude that the prevalence of insufficient free-time physical activity was high among public school teachers and, even after adjusting for the demographic, economic, and health variables, such outcome remained associated with bad or regular perception of balance between personal and professional life, perception that standing time affects work, low or very low perception of current ability for the physical requirements of work, temporary employment contract, and teaching other subjects other than physical education. These data partially support the hypothesis that the current structure of teacher work can hinder the practice of free-time physical activity. In addition, they also draw attention to the need to consider the factors related to work on actions to promote physical activity in this population

## Footnotes

a Organisation for Economic Cooperation and Development. TALIS 2013 results: an international perspective on teaching and learning. Paris: OECD; 2014. https://doi.org/10.1787/9789264196261-en

b Ministério da Saúde (BR), Secretaria de Vigilância em Saúde, Departamento de Vigilância de Doenças e Agravos não Transmissíveis e Promoção da Saúde. Vigitel Brasil 2014: vigilância de fatores de risco e proteção para doenças crônicas por inquérito telefônico. Brasília (DF); 2015.

c Ministério da Saúde (BR), Secretaria de Vigilância em Saúde, Departamento de Vigilância de Doenças e Agravos não Transmissíveis e Promoção da Saúde. Vigitel Brasil 2014: vigilância de fatores de risco e proteção para doenças crônicas por inquérito telefônico. Brasília (DF); 2015.

d Instituto Brasileiro de Geografia e Estatística. Pesquisa Nacional por Amostra de Domicílios: síntese de indicadores 2014. Rio de Janeiro; 2015.
